# Ectopic decidua of the appendix: a case report

**DOI:** 10.1186/s40792-021-01204-9

**Published:** 2021-05-11

**Authors:** Manabu Kaneko, Hiroaki Nozawa, Hirofumi Rokutan, Koji Murono, Tetsuo Ushiku, Soichiro Ishihara

**Affiliations:** 1 Department of Surgical Oncology, Faculty of Medicine, The University of Tokyo, 7-3-1 Hongo, Bunkyo-ku, Tokyo, 113-8655 Japan; 2 Department of Pathology, Faculty of Medicine, The University of Tokyo, Tokyo, Japan

**Keywords:** Ectopic decidua, Appendicitis, Pregnancy, Surgery

## Abstract

**Background:**

Ectopic decidua is the presence of decidual tissue outside the uterus. Ectopic decidua of the appendix is a rare entity that can present with abdominal symptoms mimicking appendicitis. We report a case of a 39-year-old female patient at 27 weeks gestational age with a 2-day history of right lower quadrant abdominal pain.

**Case presentation:**

The patient was referred to our hospital with suspicion of either acute appendicitis or threatened rupture of the uterus, the latter of which was considered unlikely following close examination. Therefore, she underwent emergency appendectomy via laparotomy. Microscopic examination revealed decidual tissue with myxoid degeneration in the subserosal layer of the tip side of the appendix, without endometriosis, which was compatible with ectopic decidua (deciduosis).

**Conclusions:**

Because it is extremely difficult to distinguish ectopic decidua of the appendix from acute appendicitis, even with various imaging modalities, we should be aware that ectopic decidua of the appendix is a differential diagnosis for acute appendicitis in pregnant women.

## Introduction

Normal decidua consists of endometrial stromal cells transformed during pregnancy in response to ovarian and placental hormones, specifically progesterone. Ectopic decidua is the presence of decidual tissue outside the uterus; i.e., cervix, ovary, and fallopian tube, peritoneal surface, appendix, bladder, small intestine, large intestine, mesentery, and lymph nodes, that is usually related to pregnancy [1]. Ectopic decidua of the appendix is a rare entity that can present with abdominal symptoms mimicking appendicitis.

The prevalence of acute appendicitis during pregnancy is 0.05–0.13% [2]. Acute appendicitis is the most common non-obstetric surgical emergency during pregnancy, followed by cholecystitis, pancreatitis, and bowel obstruction [3, 4]. In addition, acute appendicitis is the most common cause of non-obstetric surgical intervention performed during pregnancy, accounting for 25% of non-obstetric surgical interventions during pregnancy [5, 6].

It is extremely difficult to distinguish between ectopic decidua of the appendix and acute appendicitis, clinically. We herein report a case of ectopic decidua of the appendix in a 39-year-old female patient at 27 weeks gestational age with symptoms of suspected acute appendicitis who underwent appendectomy, which permitted a pathological diagnosis.

## Case report

**Fig. 1:**
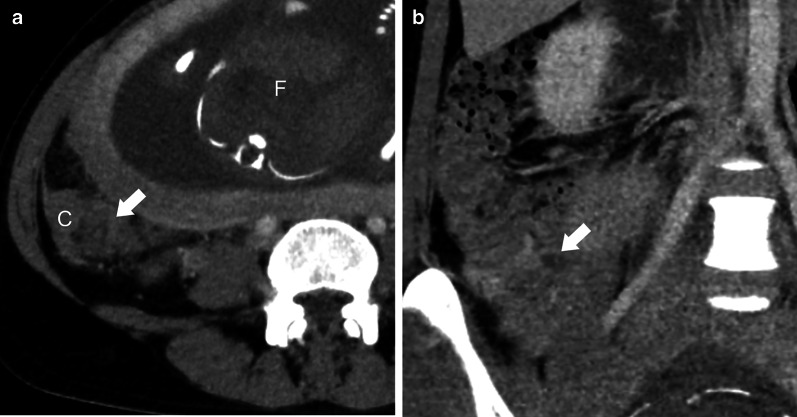
Computed tomography. **a** Axial view showing increased attenuation in fat (arrow) in the area close to the cecum (C: cecum). ‘F’ indicates the fetus. **b** Coronal view showing a swollen appendix measuring 10 mm in diameter (arrow) medial to the cecum. **c** Sagittal view showing that normal appearance of the uterus in pregnancy without findings suggestive of intrauterine infection

A 39-year-old female patient at 27 weeks gestational age was referred to our hospital with a 2-day history of right lower quadrant abdominal pain. She had no remarkable medical history. Physical examination revealed that the patient’s vital signs, including body temperature, were within normal limits, and slight tenderness was detected in the right lower abdomen, with a palpable uterus measuring > 20 cm in diameter. Laboratory tests showed low hemoglobin and albumin concentrations (9.6 g/dL and 3.0 g/dL, respectively), elevated C-reactive protein (CRP) concentration (41.3 mg/L), and slightly elevated white blood cell count (8600/µL). Ultrasonography (US) revealed a fetus that was normally developed for the gestational age and no signs of threatening uterine rupture. Abdominal computed tomography (CT) axial scans showed increased attenuation in fat in the area close to the cecum (Fig. 1a). A coronal view revealed that a luminal structure medial to the cecum appeared to be a swollen appendix 10 mm in diameter (Fig. 1b). The uterus looked normal for week 27 of pregnancy, and there were no findings suggestive of intrauterine infection (Fig. 1c). Although it was difficult to make a definitive diagnosis by imaging studies, acute appendicitis was highly suspected. After written informed consent was obtained from the patient and her family, emergency surgery was performed that day.

**Fig. 2:**
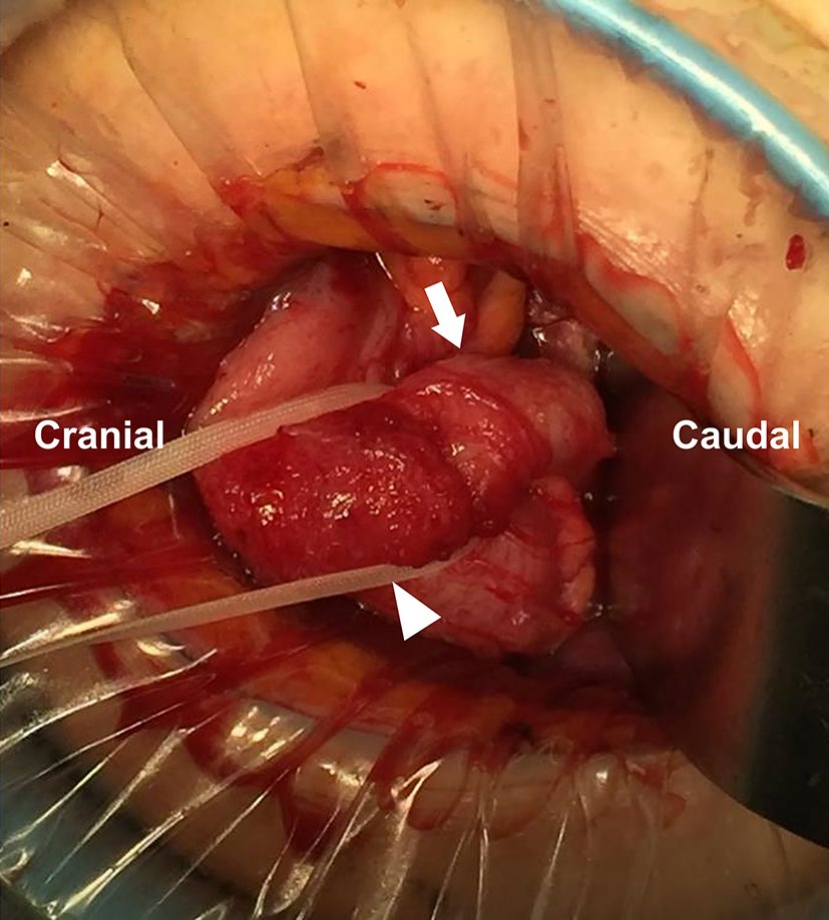
Intraoperative findings. The arrow and arrowhead indicate the body and root of the appendix, respectively

After US-guided marking, a skin incision was made just ventral to the location of the appendix. The swollen appendix covered with the omentum was identified in the space between the cecum and the distended uterus. After bloody ascites was drained, the root of the appendix was ligated first and dissected (Fig. 2). Subsequently, adhesion between the appendix and surrounding tissue was detached, and the appendix was removed.

**Fig. 3:**
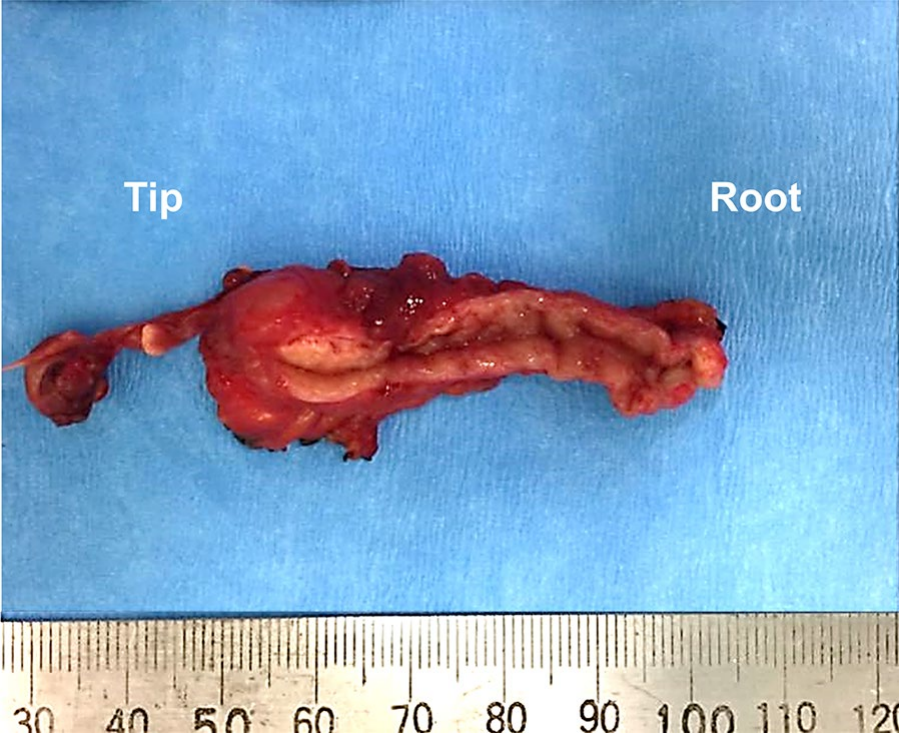
Macroscopic findings of the specimen. The size was 5.7 × 2.4 × 1.2 cm, and the tip side of the appendix was edematous. No perforation or neoplastic lesions were evident

**Fig. 4:**
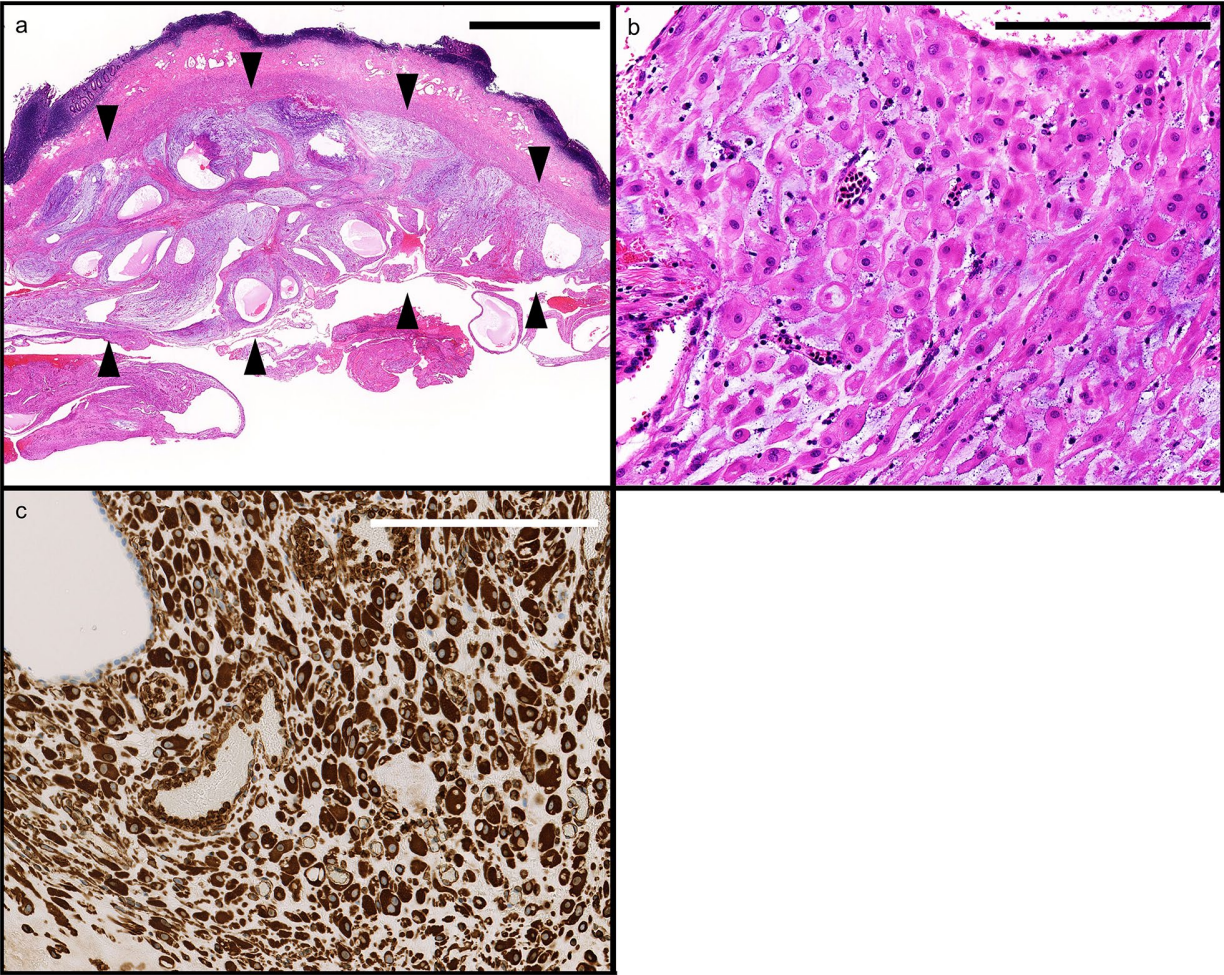
Microscopic findings. **a** Area of deciduosis, accompanied by myxoid degeneration, was widely seen in the subserosal layer (area marked by arrowheads) of the tip side (bar: 2.5 mm). **b** In a high power magnification, deciduosis is composed of large polygonal cells with abundant eosinophilic cytoplasm and centrally placed uniform nuclei, characteristic of decidual cells (bar: 2.5 µm). **c** Immunohistochemical evaluation showing diffuse positivity for vimentin in the decidual cell cytoplasm (bar: 250 µm)

Macroscopically, the resected appendix measured 5.7 × 2.4 × 1.2 cm in size with the tip side being edematous and thickened. No perforation or neoplastic lesions were observed (Fig. 3). Histologically, inflammation was scarce from the mucosa to the submucosa, and the wall structure was preserved. Decidual reaction with myxoid degeneration was widely observed in the subserosal layer on the tip side (Fig. 4a), accompanied by mild hemorrhage and focal neutrophil accumulation. Stromal cells with decidual change were characterized by abundant eosinophilic cytoplasm and centrally placed uniform nuclei (Fig. 4b). These cells were immunohistochemically positive for vimentin (Fig. 4c), CD10, and estrogen receptor. No endometriosis was found. According to these findings, we diagnosed ectopic decidua (deciduosis) of the appendix.

The patient’s postoperative course was uneventful and the CRP level dropped to 3.1 mg/L postoperative day 8. At 29 weeks gestational age, intrauterine infection was suspected owing to elevated CRP concentration (50 mg/L) and white blood cell count (18,000/µL). Thus, she underwent urgent cesarean section, and the baby was delivered safely. The cause of the elevated CRP level before appendectomy in our patient could not be clearly determined, although latent intrauterine infection was one of the possible causes.

## Discussion

Ectopic decidua is commonly localized on serosal surfaces of the pelvic organs and is incidentally detected in surgical specimens or a discrete nodule or mass found during cesarean section. In a study of 307 consecutive cesarean sections, macroscopic decidua was found in 31 (10.1%) cases [7]. Ectopic decidua with glands is subclassified as decidualized endometriosis, whereas, without glands, the condition is called deciduosis. De novo development from submesothelial stroma or pre-existing endometriosis can contribute to ectopic decidual transformation during pregnancy [8]. In addition, there are a small number of cases of ectopic decidua in non-pregnant or post-menopausal women, in whom an organizing corpus luteum, ovarian stromal cells, and adrenal-derived progesterone may be involved [1, 8, 9]. In our search using “decidua or deciduosis”, “appendix” and “pregnancy as keywords for case reports or case series of ectopic decidua of the appendix written in English in PubMed from 1966 to October 2020, 23 cases were reported to date (Table 1) [8–15]. The reported age and onset ranged from 18–40 years and 18–40 weeks gestational age. Our patient was typical, considering these characteristics. Deciduosis of the appendix occurs in a minority of patients in pregnancy (Table 1).

**Table 1:** Reported cases of ectopic decidua of the appendix

Author [reference no.]	Year	Number of patients	Patient age (years)	Gestational age (weeks)	CRP at diagnosis (mg/L)	Accompanying glands
Zaystev [9]	1987	1	33	29	NA	NA
Suster [8]	1990	6	18–40	26–40	NA	NA
Silvestrini [11]	1995	1	28	21	NA	Present
Lesaffer [12]	2009	2	19, 23	19, 24	NA	Present in 2
Chai [10]	2016	4	22–36	20–26	NA in 2, 12, < 5	NA
Murphy [13]	2016	1	31	18	9.6	Present
Noor [14]	2019	7	19–39	NA	NA	Present in 5, absent in 2
Tsunemitsu [15]	2020	1	35	33	NA	Present
Our case	2020	1	39	27	41.3	Absent

*CRP* C-reactive protein, *NA* not available

Ectopic decidua is benign, typically asymptomatic, and basically not a target of treatment because the condition usually regresses 4–6 weeks postpartum. However, ectopic decidua sometimes causes pain and intraperitoneal hemorrhage [8], which is an indication for surgery, as reported in a previous study [10]. Ectopic decidua of the appendix, which can present with symptoms mimicking acute appendicitis, is uncommon. Chai et al. stated that it is difficult to distinguish between acute appendicitis and ectopic decidua of the appendix, even with magnetic resonance imaging [10].

Conservative treatment of appendicitis with antibiotics in pregnant woman has recently gained attention as an alternative treatment option [16]; however, efficacy and safety of non-operative management with antibiotics in pregnant patients remains to be elucidated. Delaying surgical intervention for more than 24 h after symptom onset increases the risk of perforation [17]. The perforation rate of appendicitis is 14–43% in pregnant women, which is higher than the rate in non-pregnant women [18], and the fetal loss rate in pregnant women with appendicitis increases from 3–5% to 36%, with perforation [10, 19]. Moreover, antibiotics aimed at treating appendicitis will not ameliorate symptoms in case of ectopic decidua of the appendix, because they may be mechanically induced by expanding decidual tissues in response to increasing sex steroid hormone levels and/or contraction of muscle wall of the appendix mediated by prostaglandins [8]. Therefore, prompt surgical removal may be desirable for appendiceal and/or periappendiceal inflammatory changes, regardless of whether a correct diagnosis can be made.

## Conclusions

Ectopic decidua of the appendix is rare and difficult to distinguish from acute appendicitis. We should know that ectopic decidua of the appendix is a differential diagnosis for acute appendicitis in pregnant women, although surgical resection remains the first choice.

## Data Availability

All data supporting this article are included in this manuscript.

## Abbreviations

US: Ultrasonography
CT: Computed tomography
